# TGF-β2 Induces Gli1 in a Smad3-Dependent Manner Against Cerebral Ischemia/Reperfusion Injury After Isoflurane Post-conditioning in Rats

**DOI:** 10.3389/fnins.2019.00636

**Published:** 2019-06-26

**Authors:** Li Peng, Chengwei Yang, Jiangwen Yin, Mingyue Ge, Sheng Wang, Guixing Zhang, Qingtong Zhang, Feng Xu, Zhigang Dai, Liping Xie, Yan Li, Jun-qiang Si, Ketao Ma

**Affiliations:** ^1^Department of Anesthesiology, First Affiliated Hospital, School of Medicine, Shihezi University, Shihezi, China; ^2^Department of Anesthesiology, First Affiliated Hospital of USTC, Division of Life Sciences and Medicine, University of Science and Technology of China, Hefei, China; ^3^Department of Physiology, School of Medicine, Key Laboratory of Xinjiang Endemic and Ethnic Diseases, Shihezi University, Shihezi, China

**Keywords:** TGF-β, Shh, isoflurane, ischemia/reperfusion injury, neuroprotection

## Abstract

Isoflurane (ISO) post-conditioning attenuates cerebral ischemia/reperfusion (I/R) injury, but the underlying mechanism is incompletely elucidated. Transforming growth factor beta (TGF-β) and hedgehog (Hh) signaling pathways govern a wide range of mechanisms in the central nervous system. We aimed to investigate the effect of the TGF-β2/Smad3 and sonic hedgehog (Shh)/Glioblastoma (Gli) signaling pathway and their crosstalk in the hippocampus of rats with ISO post-conditioning after cerebral I/R injury. Adult male Sprague-Dawley rats were subjected to middle cerebral artery occlusion (MCAO), 1.5 h occlusion and 24 h reperfusion (MCAO/R). To assess the effect of ISO after I/R injury, various approaches were used, including neurobehavioral tests, TTC staining, HE staining, Nissl staining, TUNEL staining, immunofluorescence (IF), qRT-PCR (quantitative real-time polymerase chain reaction) and Western blot. The ISO post-conditioning group (ISO group) received 1 h ISO post-conditioning when reperfusion was initiated, leading to lower infarct volumes and neurologic deficit scores, more surviving neurons, and less damaged and apoptotic neurons. IF staining, qRT-PCR and Western blot showed high expression levels of TGF-β2, Shh and Gli1 in the hippocampal CA1 of the ISO group. Phosphorylated Smad3 (p-Smad3), Patched (Ptch), and Smoothed (Smo) were also increased at protein level in the ISO group, whereas total Smad3 expression did not change in all groups. When TGF-β2 inhibitor, pirfenidone, or Smad3 inhibitor, SIS3 HCl, were administered, the expression levels of p-Smad3 and Gli1 were reduced, and surviving pyramidal neurons decreased. By contrast, the expression levels of TGF-β2 and p-Smad3 did not change significantly after pre-injection of Smo inhibitor cyclopamine, but reduced the expression levels of Shh, Ptch, and Gli1. Moreover, Gli showed the lowest expression levels with pirfenidone combined with cyclopamine. These findings indicate that the TGF-β and hedgehog signaling pathways mediate the neuroprotection of ISO post-conditioning after cerebral I/R injury, and crosstalk between two pathways at the Gli1 level.

## Introduction

Stroke is the second leading cause of death in the world (Gbd 2015 Mortality and Causes of Death Collaborators, 2016). Its high morbidity, disability, and mortality rates have become global public health problems (Feigin et al., 2014). The early recovery of blood flow is key to the therapy of cerebral ischemia. However, despite the recovery of blocked blood flow, local brain damage and dysfunction often occur, a phenomenon known as ischemia/reperfusion (I/R) injury (Eltzschig and Eckle, 2011).

Isoflurane (ISO) is a common volatile anesthetic in the clinic and protects against ischemic brain injury (Cheon et al., 2017). For example, isoflurane provides neuroprotection in ischemic brain injury by suppressing apoptosis (Zhao et al., 2016). ISO post-conditioning can ameliorate intracranial hemorrhage and infarction volume in tPA-exaggerated brain injury (Kim et al., 2015). Besides, ISO significantly enhanced the expression of HIF-1α and VEGF, and decreased the immune cell infiltration during cerebral ischemia (Taheri et al., 2014). However, the brain protection mechanism of ISO remains to be fully elucidated. Of note, studies about signal transduction pathways after ISO post-conditioning in the central nervous system are limited, thus, this is worth researching.

TGF-β2 is a member of the TGF-β beta superfamily, involved in regulating the induction, specification, survival and maturation of cell and is important in repair following brain damage (Sakamoto et al., 2003; Polazzi et al., 2009). The binding of TGF-βs to type II receptor (TβR II) activates type I receptor (TβR I) and forms a complex. Activated TβR I stimulate phosphorylation of Smad2/3, p-Smad2/3 then associates with Smad4 and they are transferred to the nucleus. In the nucleus, activated Smads integrate transcription factors and modulate various biological effects (Shi and Massague, 2003). For example, resveratrol inhibits TGF-β2-induced epithelial-to-mesenchymal transition by suppressing the Smad pathway (Chen et al., 2017). We demonstrated previously that the TGF-β1-Smad2/3 signaling pathway is involved in the neuroprotective mechanism of ISO post-conditioning in cerebral I/R injury (Wang et al., 2016).

The hedgehog pathway participates in multiple processes of embryonic development and plays a fundamental role for the normal formation and construction of the central nervous system. In canonical hedgehog signaling, Shh links to Ptch receptor in an autocrine or paracrine manner, releasing inhibitory of Ptch on Smo receptor, allowing Glis to enter the nucleus and initiate the expressions of the target genes (Alvarez-Buylla and Ihrie, 2014). Moreover, the hedgehog non-canonical pathway also induces target genes. High levels of Gli1 proteins are a well-established hallmark of the hedgehog signaling pathway activation (Varjosalo and Taipale, 2008). In recent years, attention has been paid to its close relationship with nerve tissue repair after brain injury (Zhang et al., 2017). The Shh pathway agonist is neuroprotective (Chechneva et al., 2014) and results in enhanced functional recovery, both in locomotor function and in cognitive function (Jin et al., 2017), whereas inhibition of Shh signaling exacerbates ischemic brain injury and inhibits brain remodeling (Ding et al., 2013; Zhang et al., 2013).

Previous studies have shown that the TGF-β signaling pathway interacts with the Shh/Gli signaling pathway (Ludtke et al., 2016; Farooqi et al., 2017; Gencer et al., 2017) and that Smad3 may play an important role between the two signaling pathways. Liang found that TGF-β upregulates Gli2 via a Smad3-dependent manner and induces nuclear aggregation and DNA binding of Gli2 (Liang et al., 2017). Mcmillin’s study illustrated that TGFβ-1 inhibits Gli1 through Smad3 and promotes nervous system recession (McMillin et al., 2014). Fan identified TGF-β2 as an important regulated gene for Hh and found that the downregulation of TGF-β2 expression prevented Hh signaling-dependent neuron differentiation (Fan et al., 2010). However, whether the interaction of the Shh signaling pathway and TGF-β2 after ISO post-conditioning is involved in the protective mechanism of cerebral I/R injury and how it plays a role in this mechanism have not been explored.

We envisaged that TGF-β2 can regulate the Shh pathway via the Smad3 pathway in the brain protection of ISO post-conditioning. In this study, we investigated the effect of the TGF-β2/Smad3 and Shh/Gli signaling pathways and their crosstalk in the hippocampus of rats with ISO post-conditioning after cerebral I/R injury.

## Materials and Methods

### Animals

All animals received humane care in compliance with the National Institutes of Health Guide for the Care and Use of Laboratory Animals (NIH publications number 80–23, revised in 1996). All animal procedures were executed with the consent of the Animal Experimental Committee of the First Affiliated Hospital of the Medical College, Shihezi University. One hundred and eighty adult Sprague-Dawley male rats (220–280 g) were provided by the Experimental Animal Center of Shihezi University, China.

### Model Establishment

Rats were anesthetized using ketamine hydrochloride (60 mg/kg, intraperitoneal injection). The right common carotid artery (CCA), external carotid artery (ECA), and internal carotid artery (ICA) were exposed and isolated. Next, 3-0 monofilament nylon suture was introduced through the right CCA into the ICA until resistance was encountered. It was advanced by 18–20 mm, and a silk thread was tied to the right CCA. After 1.5 h of occlusion, the filament was withdrawn (Belayev et al., 1996) Rats in the sham group were subjected to the same procedure without the filament advanced to the MCA origin. Rats that died or suffered surgical failure or subarachnoid hemorrhage were excluded from this study. The brains specimens were acquired after 24 h and utilized for further experiments.

### Drug Treatment and Animal Grouping

Rats were administered intraperitoneally with pirfenidone (250 mg/kg) (Selleck Chemicals, Houston, TX, United States) (Shihab et al., 2002; Burghardt et al., 2007), SIS3 HCl (2.5 mg/kg) (Selleck Chemicals, Houston, TX, United States) (Li et al., 2010; Liu et al., 2018) and cyclopamine (10 mg/kg) (Cayman Chemical, Ann Arbor, MI, United States) (Alvarez et al., 2011; Yu et al., 2017) 30 min before ischemia.

Rats were randomly divided into 9 groups: animals received sham operation and an equal volume of DMSO (Sham); rats received MCAO/R and an equal volume of DMSO (I/R); MCAO/R rats were treated with TGF-β2 inhibitor pirfenidone or Smo inhibitor cyclopamine (I/R + Pir, I/R + CYC); MCAO/R rats were treated with 1.5% ISO post-conditioning for 1 h after immediate reperfusion (ISO); MCAO/R rats were treated with pirfenidone, Smad3 inhibitor SIS3 HCl, cyclopamine, pirfenidone combined with cyclopamine before ISO post-conditioning (ISO + Pir, ISO + SIS3, ISO + CYC, and ISO + Pir + CYC, respectively).

### Evaluation of Neurologic Deficit Scores

To determine neurological function, the modified Longa scores were obtained at 24 h after reperfusion by an observer blinded to the experimental conditions. The rats were scored as follows: 0, no deficits; 1, difficulty in fully extending the contralateral forelimb; 2, unable to extend the contralateral forelimb; 3, mild circling to the contralateral side; 4, severe circling; and 5, falling to the contralateral side (Ding et al., 2002).

### Measurement of Infarct Volumes

Rats were decapitated and brains slices were taken at 2 mm intervals rapidly. The infarct volume was measured using 2% 2,3,5-triphenyltetrazolium chloride (TTC) (Sigma, St. Louis, MO, United States). The slices were stained for 30 min at 37°C and then fixed in 4% paraformaldehyde (PFA) (Sigma, St. Louis, MO, United States) for 24 h. The stained slices were imaged using a digital camera and analyzed by the Image-Pro Plus 6.0 software (Media Cybernetics, Silver Springs, MD, United States). The infarct volume was measured according to the method described by Swanson: Infarct volume (%) = 100% × (contralateral hemisphere volume - non-lesioned ipsilateral hemisphere volume)/contralateral hemisphere volume (Swanson et al., 1990).

### Hematoxylin and Eosin (HE) Staining

The rats were transcardially perfused with normal saline followed by 4% PFA (Sigma, St. Louis, MO, United States) at 24 h after the MCAO. Brains were removed and post-fixed in 4% PFA for 24 h and embedded in paraffin. Four-micrometer-thick sections were cut in the microtome (KEDEE, Jinhua, ZJ, China) and stained with hematoxylin and eosin (HE). The pyramidal neurons of the CA1 region were observed, as they are the most vulnerable to I/R injury and new neurons can be found after ischemic brain injury (Nakatomi et al., 2002; Oya et al., 2009).

### Nissl Staining

Paraffin sections were deparaffinized, hydrated and stained with thionine (Solarbio, Beijing, China). Histological changes of hippocampal CA1 subfield were observed by the microscope (Olympus, Tokyo, Japan) to assess brain neuronal damage (Gong et al., 2012).

### Terminal Deoxynucleotidyl Transferase dUTP Nick end Labeling (TUNEL) Staining

The TUNEL assay was performed using the *In Situ* Cell Death Detection Kit (Roche, Basel, Switzerland, Germany) according to the manufacturer’s instruction to detect apoptosis in hippocampal CA1 cells. Apoptosis index (AI) = (the number of apoptotic cells/total cells) × 100% (Wu et al., 2015).

### Immunofluorescence (IF) Staining

Paraffin sections were deparaffinized, hydrated, repaired antigen and eliminated endogenous peroxidase routinely. After blocking for 1 h with 0.3% Triton X-100 and 10% bovine serum albumin (BSA, sections were incubated with anti-TGF-β2 (1:100, Santa Cruz Biotechnology, Santa Cruz, CA, United States), anti-Shh (1:100, Santa Cruz Biotechnology, Santa Cruz, CA, United States) and anti-Gli1 (1:100, Santa Cruz Biotechnology, Santa Cruz, CA, United States) overnight at 4°C, respectively. After washing with PBS, the sections were incubated with secondary antibody (1:50, Fluorescein-Conjugated Goat anti-Mouse IgG, ZSGB-BIO, Beijing, China) for 1 h and then stained with propidium iodide solution (PI) for 5 min in the dark. Images were captured by a confocal laser scanning microscope (Olympus, Tokyo, Japan). Mean Density = (IOD SUM)/(area sum).

### Quantitative Real-Time PCR

Total RNA of the right hippocampi was extracted using the RNeasy Mini Kit (Qiagen, Duesseldorf, Germany) according to the manufacturer’s instructions and reversed into cDNA using the Revert Aid First Strand cDNA Synthesis Kit (Bioer, Hangzhou, ZJ, China). The following primers were used for amplification:

TGF-β2, 5′-GTGATTTCCATCTACAACAGTACC-3′ (forward) and 5′-TATAAACCTCCTTGGCGTAGTAC-3′ (reverse); Shh, 5′-GAACTCCGT GGCGGCCAAATC-3′ (forward) and 5′-GTCCAGGAAGGTGAGGAAGTC-3′ (reverse); Gli1, 5′-GCCAATCACAAATCAGTCTCC-3′ (forward) and 5′-TGCTCCTAACCTGCCCAC-3′ (reverse); β-Actin,5′-AGCAGATGTGGATCAGCAAG-3′ (forward) and 5′-AACAGTCCGCCTAGAAGCAT-3′ (reverse). Minimal Information for Publication of Quantitative Real-Time PCR Experiments (MIQE) Guidelines were followed in the assays. The expression of individual values were normalized to that of the β-Actin control, and then the ratio of the relative expression levels was calculated using the 2^−ΔΔCT^ method.

### Western Blot

The proteins were isolated from hippocampal tissue using lysis buffer (Beyotime, Shanghai, China), and protein concentrations were examined using a Bicinchoninic Acid (BCA) protein assay kit (Beyotime, Shanghai, China). The proteins were separated by sodium dodecyl sulfate-polyacrylamide gel electrophoresis (SDS-PAGE) and electro-transferred to polyvinylidenedifluoride (PVDF) membranes. After blocking with 5% skimmed milk, the membranes were incubated with primary antibodies for TGF-β2, Smad3, Shh, Ptch, Smo, Gli1 (1:1000, Santa Cruz Biotechnology, Santa Cruz, CA, United States), and p-Smad3 (1:1000, Cell Signaling Technology, Danvers, MA, United States) overnight at 4°C. After washing with Tris Buffered Saline with Tween-20 (TBST) for three times over, the membranes were incubated with secondary antibodies (1:20000, ZSGB-BIO, Beijing, China) at room temperature for 2 h and then treated with enhanced chemiluminescent (ECL) reagent (ThermoFisher, Waltham, MA, United States) to detect protein expression levels. The protein bands were quantitatively analyzed using the Image J software (Rawak Software Inc., Stuttgart, Germany).

### Statistical Analysis

All quantitative data are expressed as mean ± standard deviation (SD) of three independent experiments with each experiment. Normality test was applied before one-way Analysis of Variance (ANOVA) for multiple groups comparison. Analysis among multiple groups was carried out by one-way ANOVA followed by Turkey’s *post hoc* tests. Student’s *t-*test was used for two groups comparison. Statistical analyses were performed using SPSS 19.0 (Armonk, NY, United States) software and GraphPad Prism (La Jolla, CA, United States). *P* < 0.05 was considered to be statistically significant.

## Results

### Effect of ISO Post-conditioning on Infarct Volumes and Neurologic Deficit Scores in Rats With MCAO/R

**FIGURE 1:**
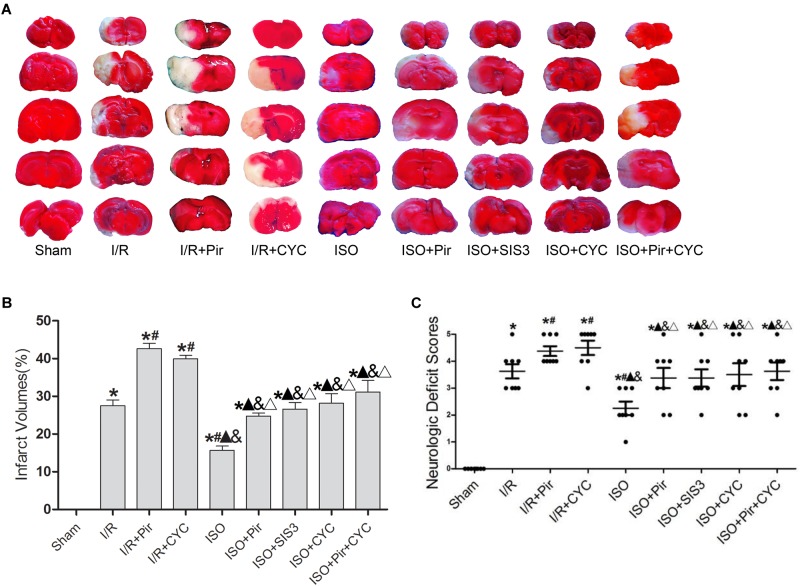
ISO post-conditioning improved infarct volumes and Neurologic Deficit Scores. **(A)** showed infarct volumes were assessed by TTC. Red represented normal tissue and white represented infarct tissues. **(B)** showed the quantitative data of infarct volumes. **(C)** showed neurological function scores with the modified Longa score. Data are shown as the mean ± SD (*n* = 8). ^∗^ *P* < 0.05 vs. Sham; # *P* < 0.05 vs. I/R; 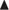 *P* < 0.05 vs. I/R + Pir; & *P* < 0.05 vs. I/R + CYC; Δ *P* < 0.05 vs. ISO.

Isoflurane treatment (1.5%) significantly decreased the infarct volumes and improved neurologic deficit scores compared with the I/R group at 24 h after MCAO/R injury in rats (15.66 ± 1.14, 2.25 ± 0.71 in the ISO group vs. 27.52 ± 1.5, 3.63 ± 0.74 in the I/R group, *P* < 0.05). However, the effects of ISO on infarct volumes and neurologic deficit scores were attenuated by inhibitors (pirfenidone, SIS3 HCl, cyclopamine, or pirfenidone combined with cyclopamine) (24.77 ± 0.82, 3.38 ± 1.06 in the ISO + Pir group; 26.6 ± 1.74, 3.38 ± 0.92 in the ISO + SIS3 group; 28.13 ± 2.58, 3.5 ± 1.2 in the ISO + CYC group; 31.1 ± 3.11, 3.63 ± 0.92 in the ISO + Pir + CYC group; *P* < 0.05). Moreover, infarct volume and neurological deficit scores were high in the MCAO/R rats treated with inhibitors (42.62 ± 1.43, 4.38 ± 0.52 in the I/R + Pir group; 39.92 ± 0.97, 4.5 ± 0.76 in the I/R + CYC; *P* < 0.05) (Figure 1 and Supplementary Tables S1, S2).

### Neuroprotective Effects of ISO Post-conditioning on the Pyramidal Cells of Hippocampal CA1 Region in Rats With MCAO/R

**FIGURE 2:**
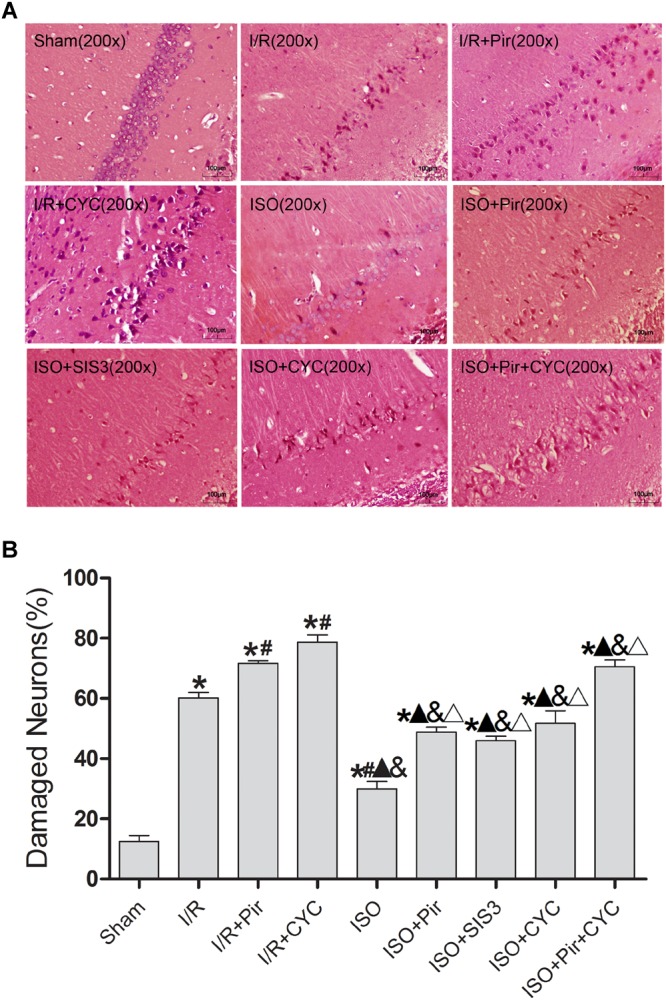
ISO post-conditioning decreased the damaged pyramidal neurons in the hippocampal CA1 region. **(A)** showed the morphology of CA1 region neurons by HE staining **(B)** showed the percentage of damaged neurons. Scale bars = 100 μm. Data are shown as the mean ± SD (*n* = 3). ^∗^ *P* < 0.05 vs. Sham; # *P* < 0.05 vs. I/R; 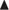 *P* < 0.05 vs. I/R + Pir; & *P* < 0.05 vs. I/R + CYC; Δ *P* < 0.05 vs. ISO.

The morphology of most hippocampal CA1 region cells in the I/R group displayed nuclear pyknosis, while that in the ISO group showed less damaged cells after HE staining (29.99 ± 2.43 in the ISO group vs. 60.16 ± 1.88 in the I/R group, *P* < 0.05). However, inhibitors (pirfenidone, SIS3 HCl, cyclopamine, or pirfenidone combined with cyclopamine) attenuated the ISO’s protective effect (48.77 ± 1.69 in the ISO + Pir group; 45.96 ± 1.51 in the ISO + SIS3 group; 51.72 ± 4.24 in the ISO + CYC group; 70.54 ± 2.26 in the ISO + Pir + CYC group; *P* < 0.05). Moreover, the percentage of damaged cells was high in the MCAO/R rats treated with inhibitors (71.69 ± 0.84 in the I/R + Pir group; 78.68 ± 2.42 in the I/R + CYC; *P* < 0.05) (Figure 2 and Supplementary Table S3).

**FIGURE 3:**
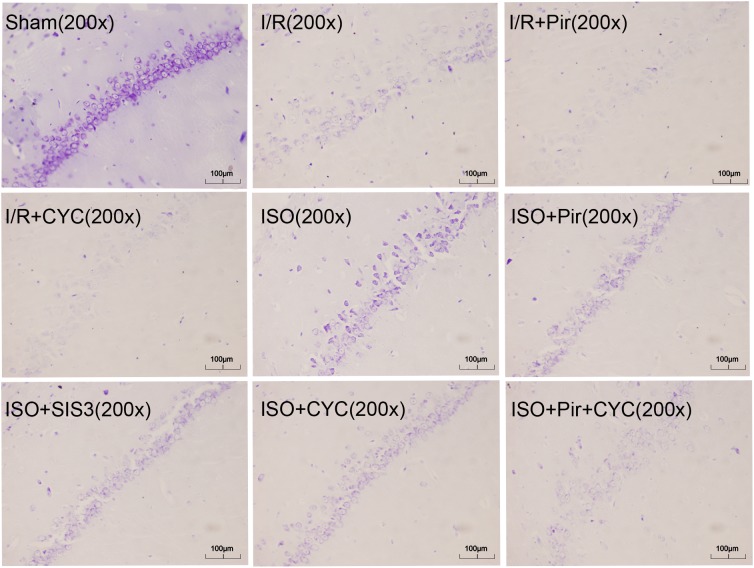
ISO post-conditioning increased the number of surviving pyramidal neurons in the hippocampal CA1 region. Nissl staining of the pyramidal neurons in the hippocampal CA1 region. Scale bars = 100 μm.

The Sham group showed abundant cells with clear borders and Nissl bodies (202.00 ± 10.39). ISO treatment (1.5%) significantly increased the surviving cells compared with the I/R group at 24 h after MCAO/R injury in rats (145.00 ± 7.48 in the ISO group vs. 85.00 ± 5.06 in the I/R group, *P* < 0.05). However, the effects of ISO on surviving pyramidal cells were attenuated by inhibitors (pirfenidone, SIS3 HCl, cyclopamine, or pirfenidone combined with cyclopamine) (102.67 ± 6.62 in the ISO + Pir group; 96.83 ± 7.41 in the ISO + SIS3 group; 99.50 ± 9.57 in the ISO+CYC group; 72.67 ± 5.99 in the ISO + Pir + CYC group; *P* < 0.05). Moreover, the number of surviving pyramidal cells in the hippocampal CA1 region were low in the MCAO/R rats treated with inhibitors (54.00 ± 4.98 in the I/R + Pir group; 50.33 ± 5.65 in the I/R + CYC; *P* < 0.05) (Figure 3, Supplementary Table S4 and Table 1).

**Table 1:** Histological grades (HG) and neuronal density (ND) in the hippocampal CA1.

Group	HG				ND
	0	I	II	III	
Sham		6			202.33 ± 10.39
I/R			5	1	85.00 ± 5.06^∗^
I/R + Pir			1	5	54.00 ± 4.98^∗^#
I/R + CYC			1	5	50.33 ± 5.65^∗^#
ISO			6		145.00 ± 7.48^∗^# 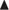 &
ISO + Pir			3	3	102.67 ± 6.62^∗^ 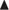 &
ISO + SIS3			3	3	96.83 ± 7.41^∗^ 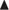 &
ISO + CYC			4	2	99.50 ± 9.57^∗^ 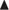 &
ISO + Pir + CYC			2	4	72.67 ± 5.99^∗^ 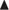 &Δ

*The standard of HG: grade 0, no cell death; grade I, scattered cell death; grade II, mass cell death; grade III, almost complete cell death. ND represented the number of surviving pyramidal neurons per 1 mm length in the CA1 hippocampus. Data are shown as the mean ± SD (n = 3). ^∗^ P < 0.05 vs. Sham; # P < 0.05 vs. I/R; 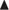 P < 0.05 vs. I/R + Pir; & P < 0.05 vs. I/R + CYC; Δ P < 0.05 vs. ISO.*

**FIGURE 4:**
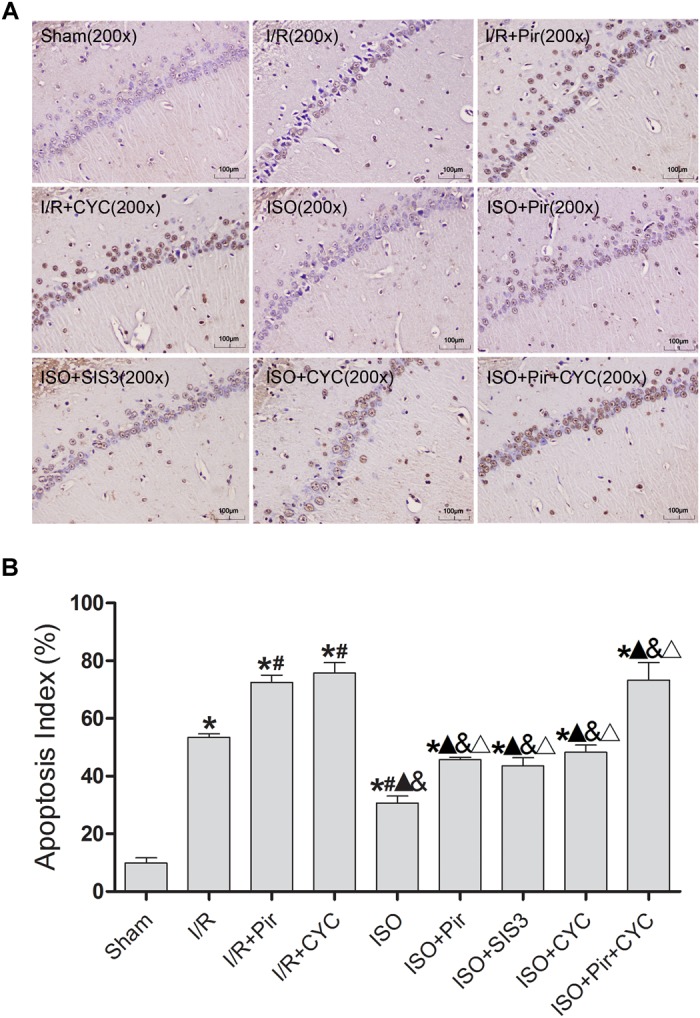
ISO post-conditioning decreased neuronal apoptosis the hippocampal CA1 region. **(A)** showed CA1 region TUNEL-positive neurons. **(B)** showed the AI in each group. Scale bars = 100 μm. Data are shown as the mean ± SD (*n* = 3). ^∗^ *P* < 0.05 vs. Sham; # *P* < 0.05 vs. I/R; 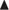 *P* < 0.05 vs. I/R + Pir; & *P* < 0.05 vs. I/R + CYC; Δ*P* < 0.05 vs. ISO.

The TUNEL-positive cells were significantly lower in the ISO group than in the I/R group (30.74 ± 2.35 vs. 53.47 ± 1.25, *P* < 0.05). However, inhibitors (pirfenidone, SIS3 HCl, cyclopamine, or pirfenidone combined with cyclopamine) attenuated the ISO’s protective effect (45.74 ± 0.80 in the ISO + Pir group; 43.54 ± 2.87 in the ISO + SIS3 group; 48.27 ± 2.55 in the ISO + CYC group; 73.31 ± 6.07 in the ISO + Pir + CYC group; *P* < 0.05). Moreover, AI was high in the MCAO/R rats treated with inhibitors (72.58 ± 2.43 in the I/R + Pir group; 75.76 ± 3.64 in the I/R + CYC; *P* < 0.05) (Figure 4 and Supplementary Table S5).

These results indicated that 1.5% ISO post-conditioning significantly ameliorated the brain I/R injury; the neuroprotective effects of ISO were remarkably inhibited with the administration of inhibitors. Of note, the injury in ISO combined with two inhibitors and I/R rats treated with inhibitors was more serious than in the other groups.

### Effect of the TGF-β2/Smad3 Signaling Pathway in the Hippocampus of Rats With ISO Post-conditioning After Cerebral I/R Injury

**FIGURE 5:**
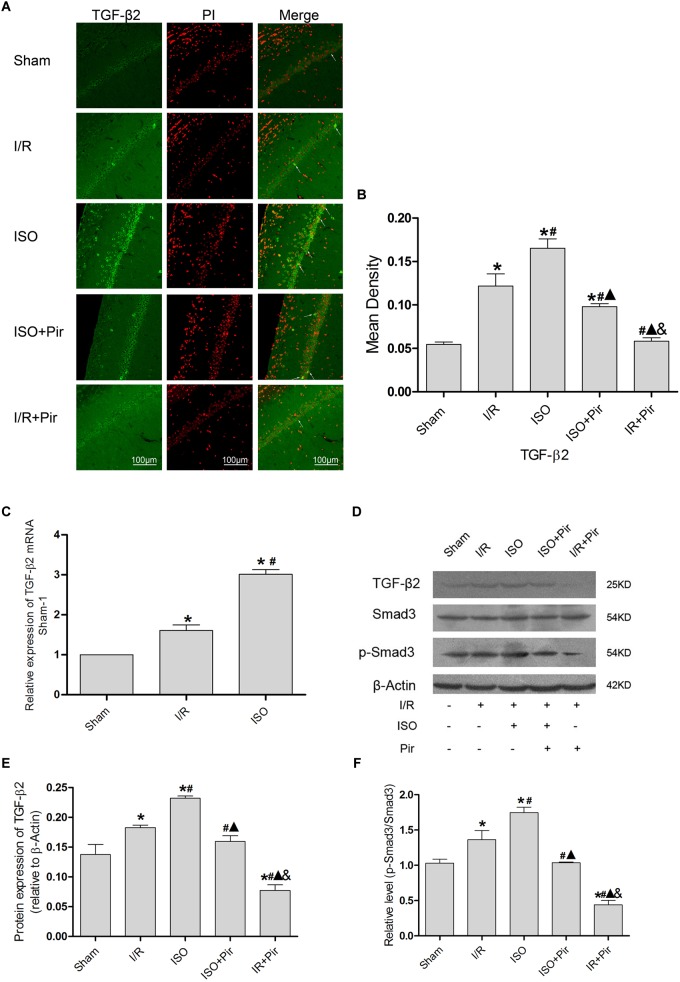
Expression of the TGF-β2/Smad3 signaling pathway in the hippocampus of rats. **(A)** showed CA1 region IF of TGF-β2. → indicates the position of TGF-β2. **(B)** showed the mean fluorescence density analysis in each group. **(C)** Expression of TGF-β2 mRNA. **(D)** Proteins expression levels of the TGF-β2/Smad3 signaling pathway. **(E)** Western blot analysis of TGF-β2. **(F)** Western blot analysis of p-Smad3/Smad3. Scale bars = 100 μm. Data are shown as the mean ± SD (*n* = 3). ^∗^ *P* < 0.05 vs. Sham; # *P* < 0.05 vs. I/R; 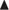 *P* < 0.05 vs. ISO; & *P* < 0.05 vs. ISO + Pir.

The IF staining showed that TGF-β2 was located in the cytoplasm (Figure 5A). IF analysis of mean density showed that the expression levels of TGF-β2 in the Sham group was relatively low (0.05 ± 0.01 in the Sham group). After 24 h reperfusion after ischemia, the expression levels of TGF-β2 significantly increased, and 1.5% ISO post-conditioning obviously increased the expression compared with the I/R group (0.16 ± 0.01 in the ISO group vs. 0.12 ± 0.01 in the I/R group, *P* < 0.05). However, pirfenidone attenuated the expression (0.10 ± 0.01 in ISO + Pir, 0.06 ± 0.01 in the I/R + Pir group, *P* < 0.05) (Figure 5B and Supplementary Table S6).

Moreover, the expression levels of TGF-β2 mRNAs were remarkably upregulated in the I/R group compared with the Sham group (1 in the Sham group vs. 1.61 ± 0.14 in the I/R group, *P* < 0.05). ISO further increased the expression levels of TGF-β2 mRNAs compared with those in the I/R group (3.01 ± 0.12 in the ISO group vs. 1.61 ± 0.14 in the I/R group, *P* < 0.05) (Figure 5C). Meanwhile, we examined the protein expression levels of the TGF-β signaling pathway (Figure 5D). After I/R injury, Western blot analysis showed that the expression levels of TGF-β2 proteins in the hippocampus were significantly higher than those in the Sham group (0.18 ± 0.01 in the I/R group vs. 0.14 ± 0.02 in the Sham group, *P* < 0.05). In addition, 1.5% ISO treatment markedly increased the expression compared with the I/R group (0.23 ± 0.01 in the ISO group vs. 0.18 ± 0.01 in the I/R group, *P* < 0.05). However, pirfenidone attenuated the expression (0.16 ± 0.01 in the ISO + Pir group vs. 0.08 ± 0.01 in the I/R + Pir group, *P* < 0.05) (Figure 5E). The change of p-Smad3/Smad3 levels were consistent with TGF-β2 (1.03 ± 0.06 in the sham group, 1.36 ± 0.13 in the I/R group, 1.75 ± 0.08 in the ISO group, and 0.44 ± 0.06 in the I/R + Pir group, *P <* 0.05) (Figure 5F and Supplementary Figure S2).

### Effect of the Shh/Gli Signaling Pathway in the Hippocampus of Rats With ISO Post-conditioning After Cerebral I/R Injury

**FIGURE 6:**
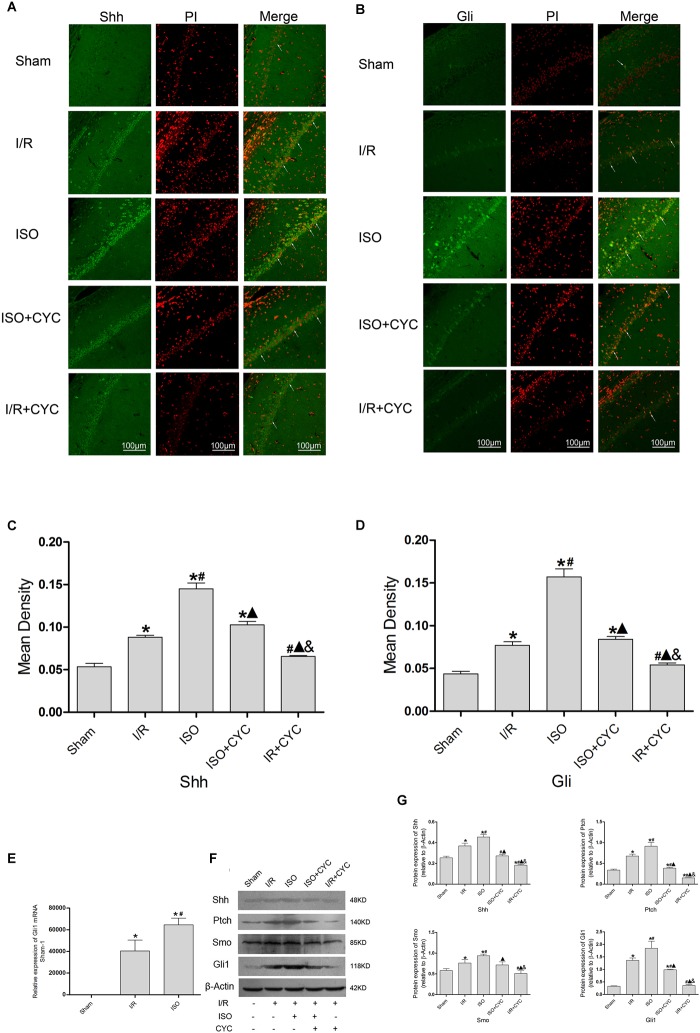
Expression of the Shh/Gli signaling pathway in the hippocampus of rats. **(A)** showed CA1 region IF of Shh. → indicates the position of Shh. **(B)** showed CA1 region IF of Gli1. → indicates the position of Gli1. **(C)** showed the mean fluorescence density of Shh in each group. **(D)** showed the mean fluorescence density analysis of Gli1. **(E)** Expression of Gli1 mRNA. **(F)** Proteins expression levels of the Shh/Gli signaling pathway. **(G)** Western blot analysis of the Shh/Gli signaling pathway. Scale bars = 100 μm. Data are shown as the mean ± SD (*n* = 3). ^∗^*P* < 0.05 vs. Sham; # *P* < 0.05 vs. I/R; 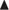 *P* < 0.05 vs. ISO; & *P* < 0.05 vs. ISO + CYC.

The IF staining showed that Shh was located in the cytoplasm (Figure 6A). Analysis of Shh’s mean fluorescence density showed that the expression levels of Shh in the Sham group were lowest (0.05 ± 0.01 in the Sham group) among all the groups. The expression levels of Shh in the I/R group at 24 h after MCAO/R injury significantly increased, and ISO application obviously increased the expression compared with the I/R group (0.15 ± 0.01 in the ISO group vs. 0.09 ± 0.01 in the I/R group, *P* < 0.05, Supplementary Table S7). However, cyclopamine attenuated the expression (0.10 ± 0.01 in the ISO + CYC group, 0.07 ± 0.01 in the I/R + CYC group, *P* < 0.05) (Figure 6C). The IF staining showed that Gli1 was involved in cytoplasm and nuclear localization. After I/R injury, Gli1 significantly increased and partially transferred to the nuclei, and ISO further promoted the nuclear translocation (Figure 6B). Fluorescence expression of Gli1 also showed the same trend with Shh (0.04 ± 0.01 in the Sham group, 0.08 ± 0.01 in the I/R group, 0.16 ± 0.01 in the ISO group, 0.08 ± 0.01 in the ISO + CYC group, 0.05 ± 0.01 in the I/R + CYC group, *P* < 0.05) (Figure 6D and Supplementary Table S8).

Moreover, the expression levels of Gli1 mRNAs were remarkably upregulated in the I/R group compared with the Sham group (1 in the Sham group vs. 40477.44 ± 9959.65 in the I/R group, *P* < 0.05). ISO further increased the expression levels of Gli1 mRNAs compared with the I/R group (64648.67 ± 6140.99 in the ISO group vs. 40477.44 ± 9959.65 in the I/R group, *P* < 0.05) (Figure 6E). Meanwhile, Western blot analysis showed that the protein expressions of Shh, Ptch, Smo, and Gli1 had similar trends in the hippocampus (Figure 6F and Supplementary Figures S3–S6). After I/R injury, the proteins were increased and ISO further promoted their expressions. In addition, the increased proteins were reduced by an inhibition cyclopamine (*P* < 0.05) (Figure 6G).

### Crosstalk Between TGF-β and Hedgehog Signaling Pathway in the Hippocampus of Rats With ISO Post-conditioning After Cerebral I/R Injury

**FIGURE 7:**
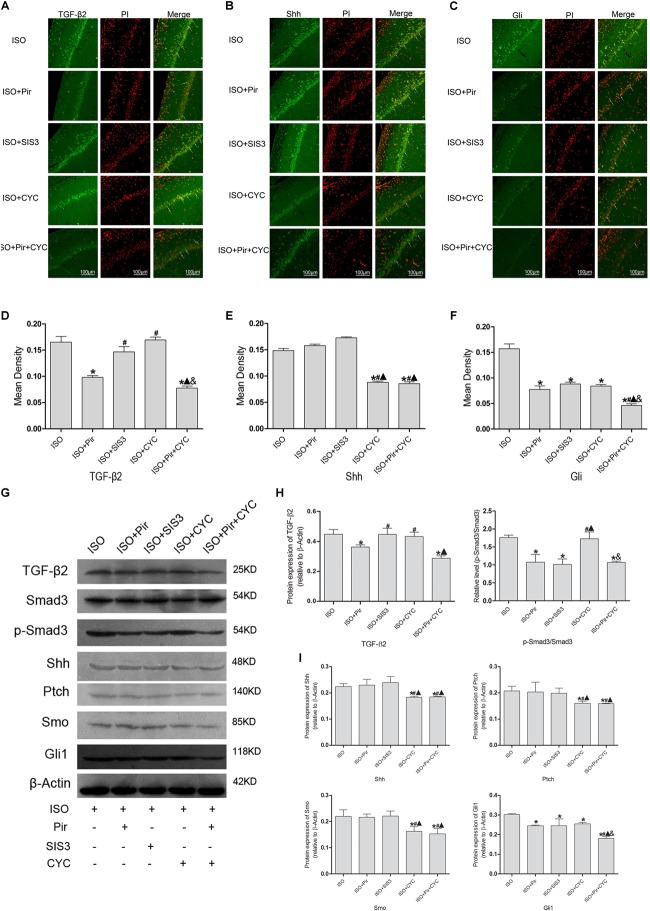
Expression of the TGF-β2/Smad3 and Shh/Gli signaling pathway in the hippocampus of rats with ISO post-conditioning after cerebral I/R injury. **(A)** showed CA1 region IF of TGF-β2. → indicates the position of TGF-β2. **(B)** showed CA1 region IF of Shh. → indicates the position of Shh. **(C)** showed CA1 region IF of Gli1. → indicates the position of Gli1. **(D)** showed the mean fluorescence density of TGF-β2. **(E)** showed the mean fluorescence density analysis of Shh. **(F)** showed the mean fluorescence density analysis of Gli1. **(G)** Proteins expression levels of the TGF-β2/Smad3 and Shh/Gli signaling pathways. **(H)** Western blot analysis of the TGF-β2/Smad3 signaling pathway. **(I)** Western blot analysis of the Shh/Gli signaling pathway. Scale bars = 100 μm. Data are shown as the mean ± SD (*n* = 3). ^∗^ *P* < 0.05 vs. ISO; # *P* < 0.05 vs. ISO + Pir; 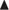 *P* < 0.05 vs. ISO + SIS3; & *P* < 0.05 vs. ISO + CYC.

The IF staining and mean fluorescence density analysis of TGF-β2 in the hippocampus of rats with ISO post-conditioning after cerebral I/R injury showed that TGF-β2 expression levels were reduced by pirfenidone, an inhibitor of TGF-β2, compared with the ISO group (0.17 ± 0.01 in the ISO group vs. 0.10 ± 0.01 in the ISO + Pir group, *P* < 0.05), whereas treatment with SIS3 HCl (a Smad3 inhibitor) or cyclopamine (a SMO inhibitor) did not significantly reduce the expression levels (0.15 ± 0.01 in the ISO + SIS3 group, 0.17 ± 0.01 in the ISO + CYC group, *P* > 0.05). Moreover, no difference was observed between the expression levels when pirfenidone combined with cyclopamine or pirfenidone alone was administered (0.10 ± 0.01 in the ISO + Pir group vs. 0.08 ± 0.01 in the ISO + Pir + CYC group, *P* > 0.05) (Figure 7A,D). The protein level of TGF-β2 showed a similar trend with it (Supplementary Figure S7). What’s more, the relative expression level of p-Smad3/Smad3 was decreased by pirfenidone, SIS3 HCl or pirfenidone combined with cyclopamine, compared with the ISO group (1.07 ± 0.21 in the ISO + Pir group, 1.01 ± 0.16 in the ISO + SIS3 group, 1.07 ± 0.04 in the ISO + Pir + CYC group vs. 1.76 ± 0.06 in the ISO group, *P* < 0.05, Supplementary Figures S8, S9). Administered with cyclopamine had no apparent effect compared with the ISO group (1.73 ± 0.19 in the ISO + CYC group vs. 1.76 ± 0.06 in the ISO group, *P* > 0.05) (Figure 7H).

The IF staining and mean fluorescence density analysis of Shh expression levels showed no statistical difference in the ISO, ISO + Pir and ISO + SIS3 groups (0.15 ± 0.01 in the ISO group, 0.16 ± 0.01 in the ISO + Pir group, 0.17 ± 0.01 in the ISO + SIS3 group, *P* > 0.05). However, with cyclopamine or pirfenidone combined with cyclopamine treatment, the expression levels were remarkably reduced. No difference was observed between the two groups (0.09 ± 0.01 in the ISO + CYC group, 0.09 ± 0.01 in the ISO + Pir + CYC group, *P* > 0.05) (Figure 7B,E). Consistently, the stimulatory effects of ISO on Shh protein and expression levels were inhibited by cyclopamine or pirfenidone combined with cyclopamine (0.22 ± 0.01 in the ISO group,0.23 ± 0.01 in the ISO + Pir group, 0.24 ± 0.01 in the ISO + SIS3 group, 0.18 ± 0.01 in the ISO + CYC group, 0.19 ± 0.01 in the ISO + Pir + CYC group, *P* < 0.05) (Figure 7I).

The IF staining and mean fluorescence density analysis showed that the expression levels of Gli1 were decreased by pirfenidone, SIS3 HCl, cyclopamine, or pirfenidone combined with cyclopamine, compared with the ISO group. Moreover, the ISO + Pir + CYC group had the lowest expression levels of Gli1 (0.16 ± 0.01 in the ISO group, 0.08 ± 0.01 in the ISO + Pir group, 0.09 ± 0.01 in the ISO + SIS3 group, 0.08 ± 0.01 in the ISO + CYC group, 0.05 ± 0.01 in the ISO + Pir + CYC group, *P* < 0.05) (Figure 7C,F). Protein expression levels of Gli1 also showed the same trend (0.30 ± 0.01 in the ISO group, 0.25 ± 0.01 in the ISO + Pir group, 0.25 ± 0.01 in the ISO + SIS3 group, 0.26 ± 0.01 in the ISO + CYC group, 0.18 ± 0.01 in the ISO + Pir + CYC group, *P* < 0.05) (Figure 7I).

Furthermore, the results of Western blot analysis indicated that the expression levels of Ptch and Smo in the hippocampus of rats with ISO post-conditioning after cerebral I/R injury had a similar trend to those of Shh (Figure 7G,I, Supplementary Table S9 and Supplementary Figures S10–S12).

## Discussion

Cerebral I/R injury is the main pathological mechanism leading to brain ischemic injury. Therefore, the prevention and treatment of reperfusion injury is key in the treatment of ischemic stroke. We demonstrated previously that 1.5% ISO post-conditioning provided the best neuroprotection in rats after focal cerebral I/R injury and DMSO exerted no effects on I/R injury (Wang et al., 2016).

Moreover, based on our current findings, we observed that ISO post-conditioning can significantly improve neurobehavioral tests and brain infarct volumes after I/R injury. The neuroprotective effect of ISO post-conditioning was also observed in HE, Nissl, TUNEL, and IF staining. However, the exact mechanisms warrant further investigations.

*In vitro* experiments proved that the proliferation and differentiation of hippocampal neurons are regulated by TGF-β2 via the SMAD pathway (Lu et al., 2005). *In vivo* studies demonstrated that TGF-β2 participates in the pathological process after cerebral I/R injury and sustains the expression of neurons (Hu et al., 2008). TGF-β2 may directly inhibit certain genes that initiate apoptosis after ischemic injury to protect neurons ^(^Dhandapani and Brann, 2003). Thus, we first analyzed the role of the canonical TGF-β signaling pathway in the hippocampus of rats with ISO post-conditioning after cerebral I/R injury. TGF-β2 and phosphorylated Smad3 were upregulated after I/R injury and further increased by ISO post-conditioning, leading to lower infarct volumes and neurologic deficit scores, more surviving neurons, and less damaged and apoptotic neurons. Pirfenidone treatment prevented the upregulation and canceled the neuroprotective effects. Our results proved that the TGF-β2/Smad3 signaling pathway played an important role in the neuroprotection of ISO post-conditioning.

We next aimed to analyze whether the Shh/Gli signaling pathway is also involved in this process. One study showed that the expression levels of Shh in hippocampal neurons were significantly upregulated after ischemic brain injury, and that cyclopamine could inhibit the proliferation of hippocampal granule neural stem cells (Sims et al., 2009). In another study, exogenous administration of Shh improved the behavioral score of rats with cerebral I/R, reduced the volumes of cerebral infarction, and promoted the angiogenesis of ischemic peripheral tissues and the colonization of neural stem cells, and cyclopamine abrogated the neuroprotective effect of Shh (Huang et al., 2013). Our results were consistent with these studies and showed that ISO can also further increase the *in vivo* expression of the Shh/Gli signaling pathway and cyclopamine can inhibit the increased expression to abrogate neuroprotective effect of ISO. Therefore, our study indicated that the neuroprotective effect of ISO is related to the Shh/Gli signaling pathway.

Finally, considering the close contact between the TGF-β and Hedgehog signaling pathways, we explored the crosstalk between these two pathways in the neuroprotection of ISO post-conditioning after cerebral I/R injury.

In our study, we investigated the effect of TGF-β signaling pathway inhibitors on the expression of Shh, Ptch, Smo, and Gli1 in the hippocampus of rats with ISO post-conditioning. The application of pirfenidone decreased the expression levels of Gli1, but did not affect Shh, Ptch, and Smo. With SIS3 HCl (an inhibitor of Smad3) treatment, the expression levels of TGF-β2, Smad3, Shh, Ptch, and Smo did not change, but those of phosphorylated Smad3 and Gli1 were reduced. These similar effects of pirfenidone and SIS3 HCl on hedgehog signaling indicated that via Smad3 pathway, TGF-β2 can affect Gli1, but not Shh, Ptch, or Smo (Supplementary Figure S1).

Meanwhile, we also used cyclopamine, an inhibitor of the hedgehog signaling pathway, to assess the expression levels of TGF-β and hedgehog signaling. Cyclopamine decreased the expression of hedgehog signaling, Shh, Ptch, Smo, and Gli, however, it did not inhibit TGF-β2, phosphorylated Smad3, or total Smad3. These results suggested that cyclopamine cannot affect the TGF-β2/Smad3 signaling pathway.

Additionally, with the administration of pirfenidone combined with cyclopamine, the expression levels of TGF-β2 and p-Smad3 were inhibited, but did not differ from the ISO + Pir group, whereas Shh, Ptch, and Smo showed no statistical significance compared with the administration of cyclopamine alone. However, the expression levels of Gli1 were lowest among all the groups. These results further illustrated that cyclopamine cannot affect the TGF-β2/Smad3 signaling pathway and that Gli1 is a downstream mediator of both TGF-β and hedgehog signaling pathways and is regulated by them. In other words, the TGF-β and hedgehog signaling pathway converged to the key protein Gli1, and activation of Gli1 expression by TGF-β2 does not involve the Ptch/Smo axis.

The TGF-β and hedgehog signaling pathways govern a wide range of mechanisms in central nervous system development, including neuronal differentiation and survival and stem cell behavior, during embryogenesis and in adulthood (Rodriguez-Martinez and Velasco, 2012; Alvarez-Buylla and Ihrie, 2014; Kandasamy et al., 2014). In fact, each family of ligands can potentially activate several intracellular pathways, and individual pathways can, in some cases, be activated by different families of ligands, hence crosstalk occurs between pathways (Hebert, 2013).

Therefore, taken together with our study, there are numbers of cases that indicated crosstalk occurred between the TGF-β and Shh pathways. TGF-β induces Gli1 and Gli2 in a Smad3-dependent manner in epithelial and mesenchymal cell lines (Steinway et al., 2014), dermal fibroblasts (Dennler et al., 2009), osteolytic Langerhans cells (Alexandrescu et al., 2012), breast carcinoma cells (Dennler et al., 2007), and pancreatic ductal adenocarcinoma cells (Nolan-Stevaux et al., 2009). Furthermore, the TGF-β/Smad/Gli2 axis has been suggested to be essential for cancer metastasis (Javelaud et al., 2011). Recent studies have identified TGF-β as a potent transcriptional regulator of Gli2, resulting in subsequent Gli1 activation independently of the Hh signaling cascade (Javelaud et al., 2012). The possible cause of this phenomenon is that hypoxia can trigger other factors (e.g., TGF-β, KRAS, or RTK), bypassing Smo to activate Gli1 directly (Lei et al., 2013).

While TGF-β likely contributes to some of the biological effects of Hh, it is also highly likely that the opposite is true. TGF-β induces Shh significantly in cultured alveolar epithelial cells, whereas Shh induces TGF-β in lung fibroblasts (Hu et al., 2015). Hh inhibitors do not affect TGF-β target gene expression in reticular fibroblasts, and TGF-β inhibition does not prevent Hh target gene induction in papillary fibroblasts (Lichtenberger et al., 2016). Consequently, the responses depend on cell lineages responding to different paracrine signals.

In addition to the Smad3-dependent activation, it is plausible that the non-canonical TGF-β pathway is capable of inducing Gli expression. PKA blockade may contribute to increasing the pool of full-length activator Gli proteins in the cell, thus allowing a possible Hh response (Pierrat et al., 2012). Basic fibroblast growth factor activation of the ERK pathway also stimulates Gli1 activity through its NH2-terminal domain (Riobo et al., 2006). These topics require further research in the future. Moreover, the anesthetic Ketamine also showed the neuroprotective effects (Shibuta et al., 2006) and further ameliorated the cerebral I/R injury. However, since ketamine is used for each rat, it does not affect our conclusions.

## Conclusion

In conclusion, our study demonstrates that both the TGF-β and hedgehog signaling pathways play essential roles in mediating neuroprotection of ISO post-conditioning after cerebral I/R injury, and the detailed crosstalk between the two pathways. Our findings provide vital insights into the effects of TGF-β and Shh signaling in the neuroprotective mechanism of ISO after ischemic stroke and may open new avenues in stroke therapy.

## Ethics Statement

All animal procedures were executed with the consent of the Animal Experimental Committee of the First Affiliated Hospital of the Medical College, Shihezi University. Two-month-old Sprague-Dawley male rats (220–280 g) were provided by the Experimental Animal Center of Shihezi University, China.

## Author Contributions

SW, LP, JY, and MG conceived and designed the experiments. LP, GZ, QZ, and FX conducted the experiments. ZD, LX, YL, J-qS, and KM provided assistance in experiments performing. LP and MG analyzed the data. LP, CY, and JY wrote the manuscript. All authors discussed and commented on the manuscript.

## Conflict of Interest Statement

The authors declare that the research was conducted in the absence of any commercial or financial relationships that could be construed as a potential conflict of interest.

## Supplementary Material

FIGURE S1 Graphical Abstract.

FIGURE S2 p-Smad3 in Figure 5D.

FIGURE S3 Shh in Figure 6F.

FIGURE S4 Ptch in Figure 6F.

FIGURE S5 Smo in Figure 6F.

FIGURE S6 Gli1 in Figure 6F.

FIGURE S7 TGF-β2 in Figure 7G.

FIGURE S8 Smad3 in Figure 7G.

FIGURE S9 p-Smad3 in Figure 7G.

FIGURE S10 Shh in Figure 7G.

FIGURE S11 Ptch in Figure 7G.

FIGURE S12 Smo in Figure 7G.

TABLE S1 Neurologic Deficit Scores.

TABLE S2 TTC Infarct Volumes.

TABLE S3 HE Damaged Neurons.

TABLE S4 Nissl staining Surviving Neurons.

TABLE S5 TUNEL-positive neurons.

TABLE S6 TGF-β2 Mean Density.

TABLE S7 Shh Mean Density.

TABLE S8 Gli Mean Density.

TABLE S9 WB.
