# Supplementary figures and images for: TGF-β2 Induces Gli1 in a Smad3-Dependent Manner Against Cerebral Ischemia/Reperfusion Injury After Isoflurane Post-conditioning in Rats
Source: Front Neurosci. 2019 Jun 26;13:636. doi: 10.3389/fnins.2019.00636 (PMC6608402; doi:10.3389/fnins.2019.00636)

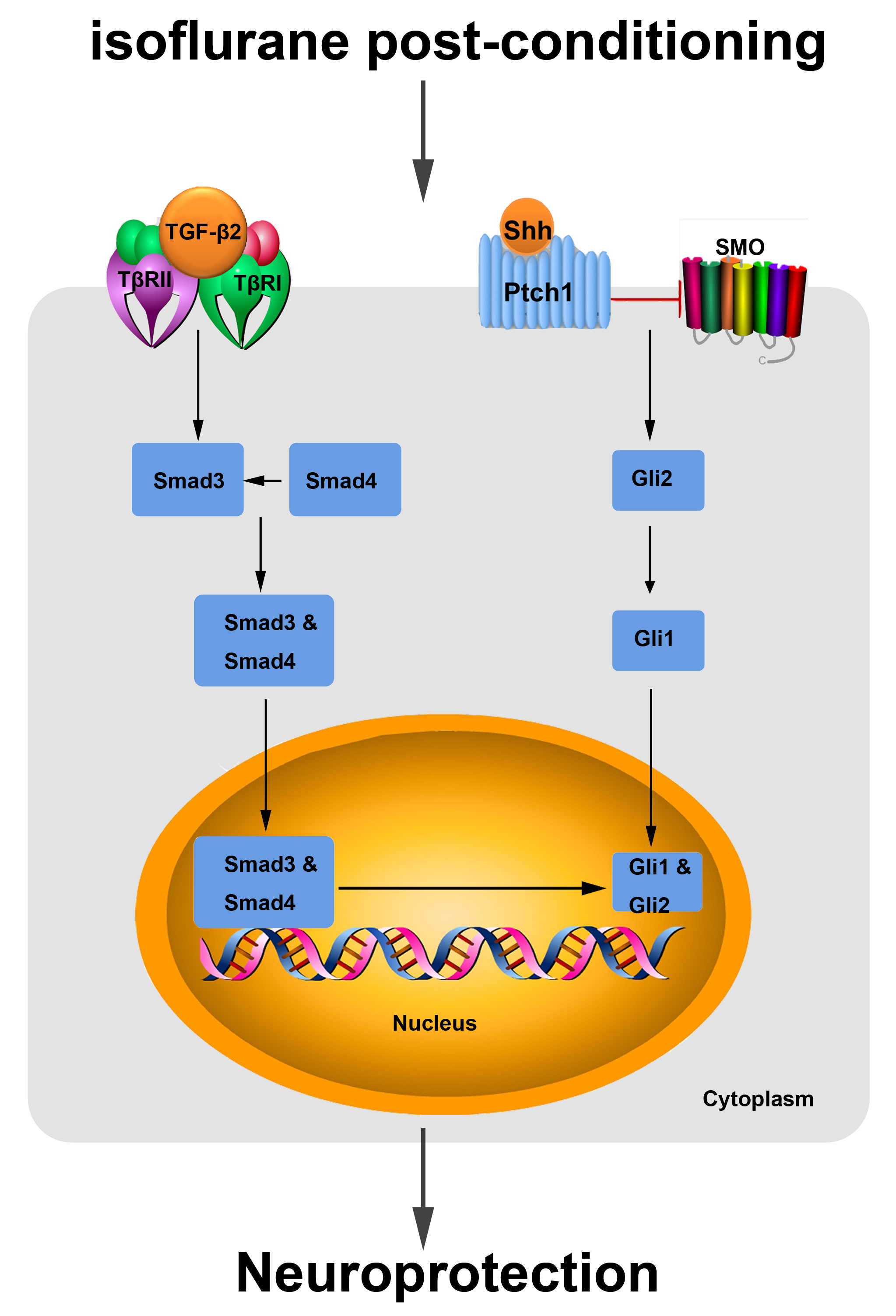

Supplement: FIGURE S1 — Graphical Abstract. [file Image_1.TIF]

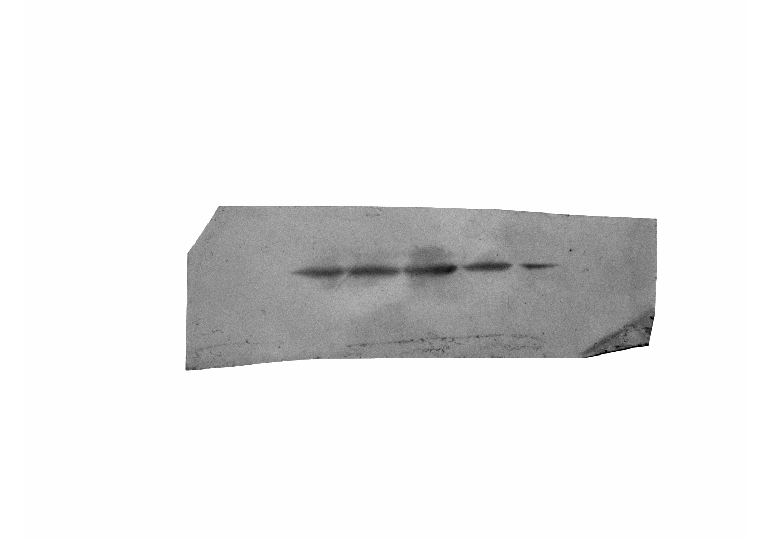

Supplement: FIGURE S2 — p-Smad3 in Figure 5D. [file Image_2.TIF]

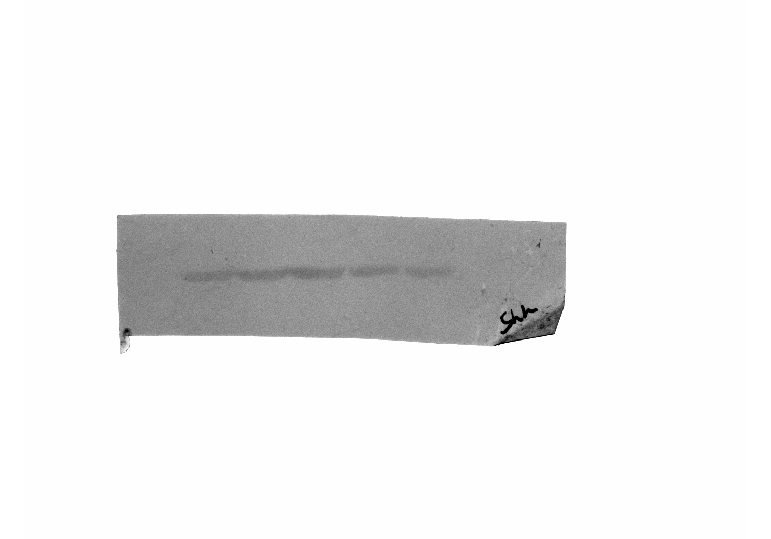

Supplement: FIGURE S3 — Shh in Figure 6F. [file Image_3.TIF]

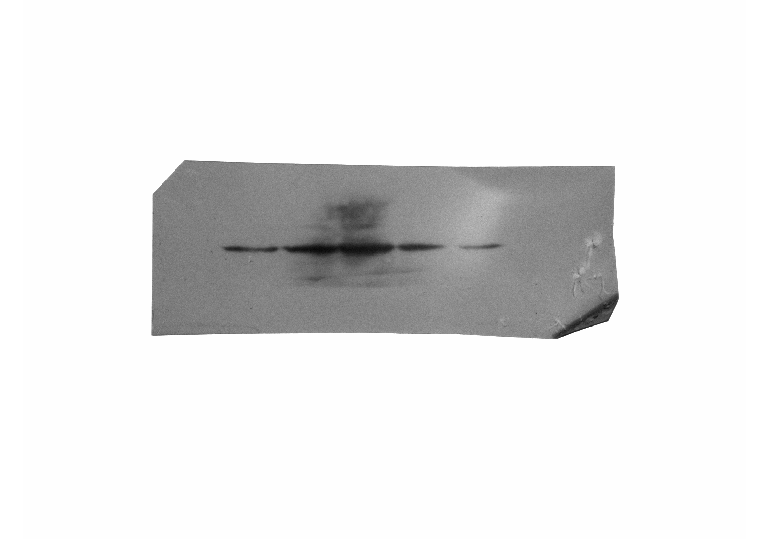

Supplement: FIGURE S4 — Ptch in Figure 6F. [file Image_4.TIF]

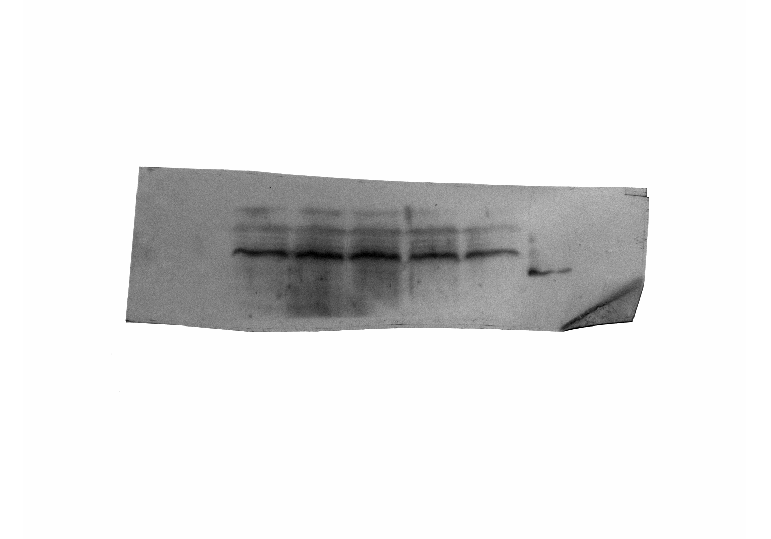

Supplement: FIGURE S5 — Smo in Figure 6F. [file Image_5.TIF]

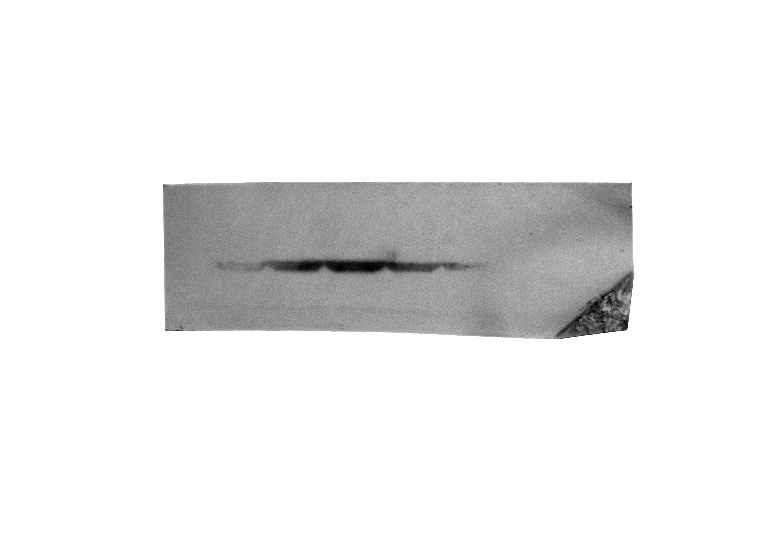

Supplement: FIGURE S6 — Gli1 in Figure 6F. [file Image_6.TIF]

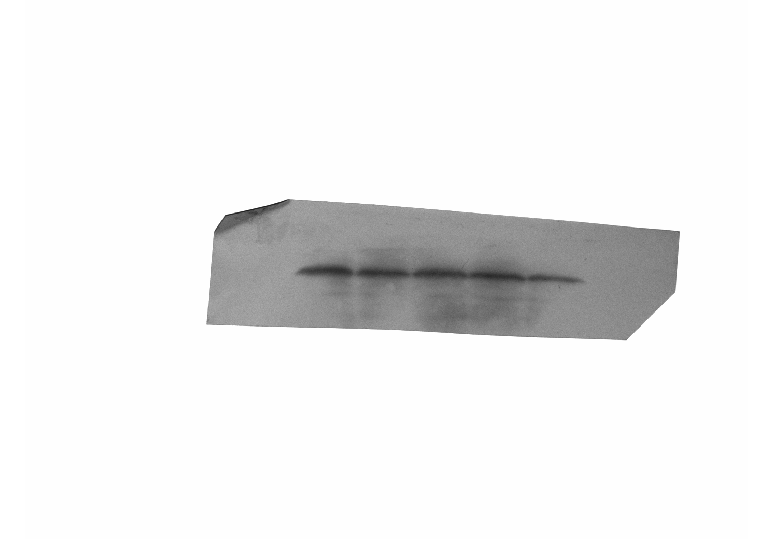

Supplement: FIGURE S7 — TGF-β2 in Figure 7G. [file Image_7.TIF]

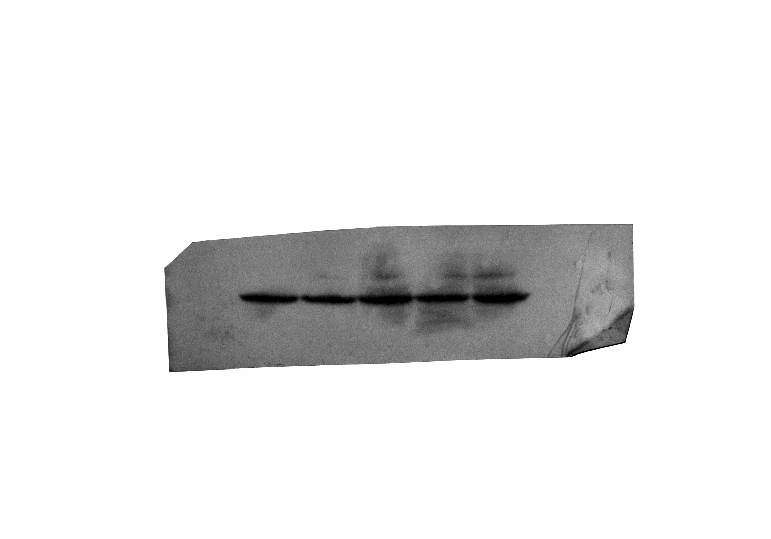

Supplement: FIGURE S8 — Smad3 in Figure 7G. [file Image_8.TIF]

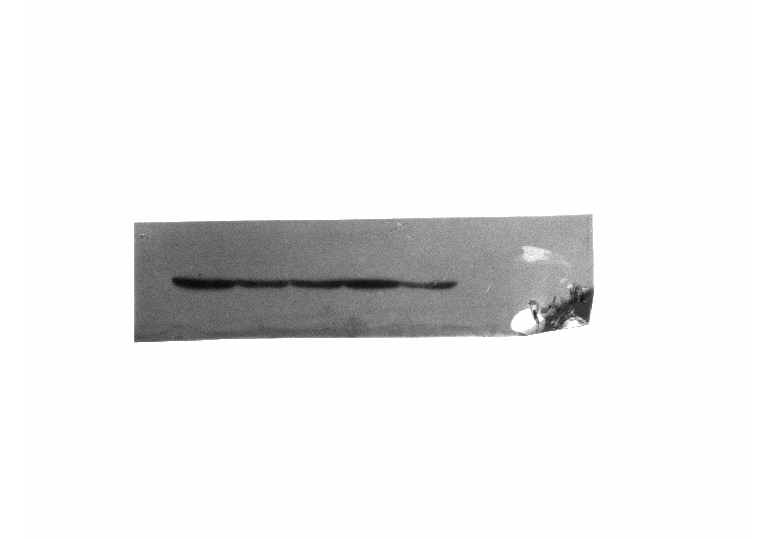

Supplement: FIGURE S9 — p-Smad3 in Figure 7G. [file Image_9.TIF]

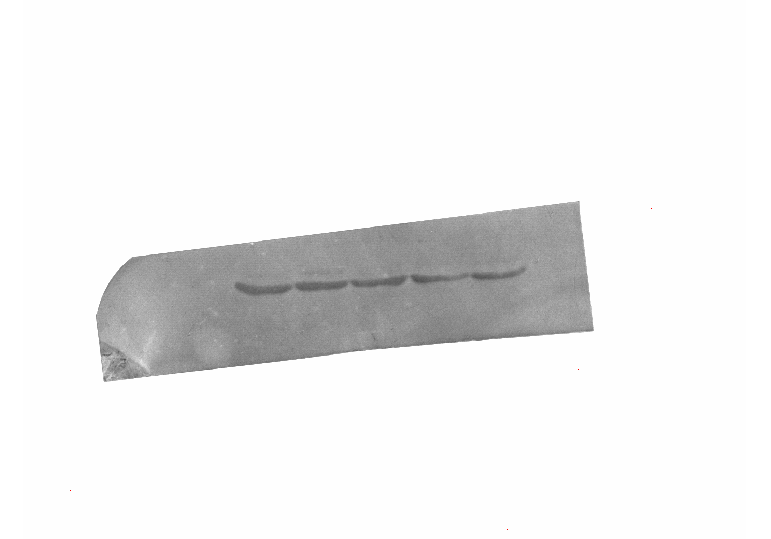

Supplement: FIGURE S10 — Shh in Figure 7G. [file Image_10.TIF]

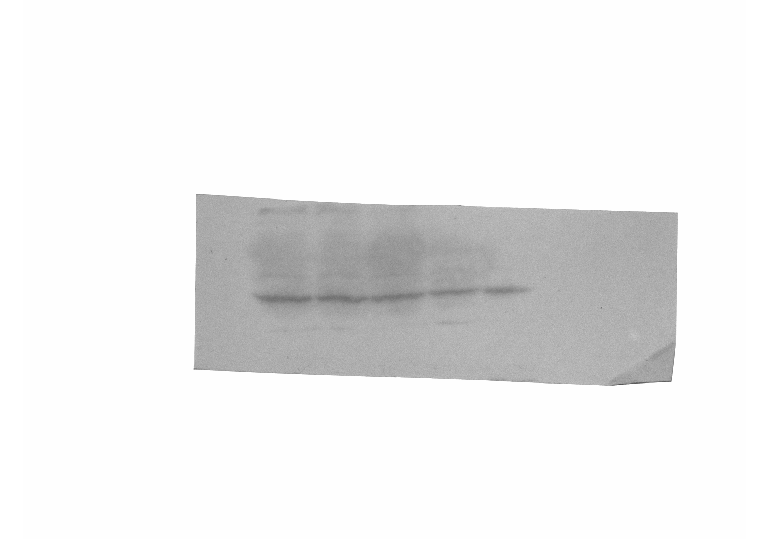

Supplement: FIGURE S11 — Ptch in Figure 7G. [file Image_11.TIF]

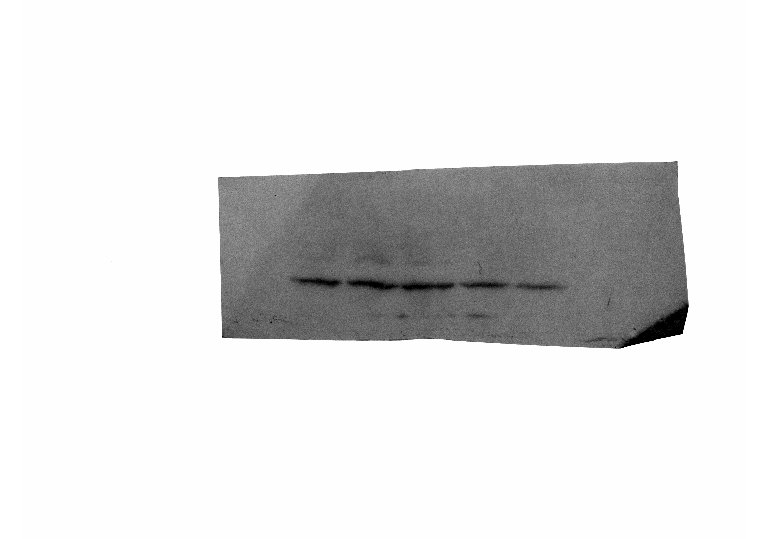

Supplement: FIGURE S12 — Smo in Figure 7G. [file Image_12.TIF]
